# Too Close for Comfort? Isotopic Niche Segregation in New Zealand’s Odontocetes

**DOI:** 10.3390/biology11081179

**Published:** 2022-08-05

**Authors:** Katharina J. Peters, Sarah J. Bury, Bethany Hinton, Emma L. Betty, Déborah Casano-Bally, Guido J. Parra, Karen A. Stockin

**Affiliations:** 1 Cetacean Ecology Research Group, School of Natural Sciences, Massey University, Auckland 0745, New Zealand; 2 School of Earth and Environment, University of Canterbury, Christchurch 8041, New Zealand; 3 Evolutionary Genetics Group, Department of Anthropology, University of Zurich, 8057 Zurich, Switzerland; 4 National Institute of Water and Atmospheric Research, Wellington 6021, New Zealand; 5 Cetacean Ecology, Behaviour and Evolution Lab, College of Science and Engineering, Flinders University, Adelaide 5001, Australia

**Keywords:** diet, dolphins, stable isotopes, nitrogen, carbon, feeding ecology, trophic relationships, SGD14

## Abstract

**Simple Summary:**

We used carbon and nitrogen stable isotopes as ecological tracers to investigate isotopic niche overlap between 21 odontocete (toothed whale) species inhabiting neritic, mesopelagic, and bathypelagic waters. Results showed a clear niche separation for the bathypelagic Gray’s beaked whales (*Mesoplodon grayi*) and sperm whales (*Physeter macrocephalus*), but high isotopic niche overlap and potential interspecific competition for neritic and mesopelagic species. This study represents the first insights into the coexistence of odontocetes in a biodiverse hotspot and provides a critical baseline for a system already undergoing ecosystem changes via ocean warming and its subsequent effect on prey abundance and distribution.

**Abstract:**

Species occurring in sympatry and relying on similar and limited resources may partition resource use to avoid overlap and interspecific competition. Aotearoa, New Zealand hosts an extraordinarily rich marine megafauna, including 50% of the world’s cetacean species. In this study, we used carbon and nitrogen stable isotopes as ecological tracers to investigate isotopic niche overlap between 21 odontocete (toothed whale) species inhabiting neritic, mesopelagic, and bathypelagic waters. Results showed a clear niche separation for the bathypelagic Gray’s beaked whales (*Mesoplodon grayi*) and sperm whales (*Physeter macrocephalus*), but high isotopic niche overlap and potential interspecific competition for neritic and mesopelagic species. For these species, competition could be reduced via temporal or finer-scale spatial segregation or differences in foraging behaviour. This study represents the first insights into the coexistence of odontocetes in a biodiverse hotspot. The data presented here provide a critical baseline to a system already ongoing ecosystem change via ocean warming and subsequent effects on prey abundance and distributions.

## 1. Introduction

An ecological niche is defined as a region in a multi-dimensional space of biotic and abiotic conditions that affect the welfare and viability of a species [1]. These dimensions can generally be divided into three main groups: temporal (i.e., diel foraging patterns or annual migrations), spatial (both horizontal and vertical), and trophic (i.e., trophic level, diet composition). The competitive exclusion principle, also known as the Gause principle or Gause’s law [2], states that two species cannot exist at constant population numbers if they are competing for the same limited resource and thus occupy the same ecological niche. To avoid competitive exclusion, co-occurring species can attain niche differentiation through resource partitioning via any of the three dimensions (temporal, spatial, or trophic) [1,2].

Knowledge of an animal’s diet is fundamental to understanding its habitat requirements, movement, and functional position in the food web [3,4,5]. Such data inform the degree of interactions between different taxa, as well as the grade of dietary specialisation and hence foraging plasticity of a species [6,7,8]. Stable isotope analysis enables an animal’s niche to be quantified using the concept of “isotopic niche” [9], with bulk carbon heavy-to-light isotopic ratios (δ^13^C) providing information on likely carbon sources relating to feeding habitat [10,11,12], and bulk nitrogen isotopic ratios (δ^15^N) informing trophic level [13,14] and foraging ecology [15,16,17,18].

Aotearoa, New Zealand is home to an extraordinarily rich marine fauna, including 50% of the world’s cetacean species [19,20]. Of these, 35 are odontocetes (toothed whales), which are apex- and mesopredators that play a crucial part in maintaining ecosystem health [20]. Due to New Zealand’s latitudinal spread and isolated geographical location, these species occupy a range of different habitats, ranging from shallow coastal and shelf areas over deep ocean trenches to subantarctic waters [21,22]. While a degree of inherent spatial segregation between taxa is expected given their different habitat requirements and foraging behaviour, many species generally overlap in their distribution [19]. To minimise interspecific competition, predators occupying the same habitat usually practise resource partitioning by exploiting available prey differently [23,24]. Alternatively, or in some cases, additionally, predators segregate spatially or temporally to avoid competition [25,26]. Besides being home to a diverse cetacean community, New Zealand is also a hotspot for cetacean stranding events, including live single and mass stranding events, as well as dead ‘beachcast’ specimens that wash ashore [27,28,29]. While many of these stranding events involve species that regularly occur in New Zealand’s Economic Exclusion Zone, occasionally even vagrant Antarctic species are recorded on New Zealand shores (New Zealand National Strandings Database, Department of Conservation).

In this study, we investigated the isotopic niche of 21 odontocete species occurring in New Zealand waters using skin samples collected from live-stranded and beach-cast individuals. We hypothesised that species with similar habitat requirements would occupy their own respective isotopic niche, thus avoiding interspecific resource competition.

## 2. Materials and Methods

### 2.1. Sample Collection

**Figure 1:**
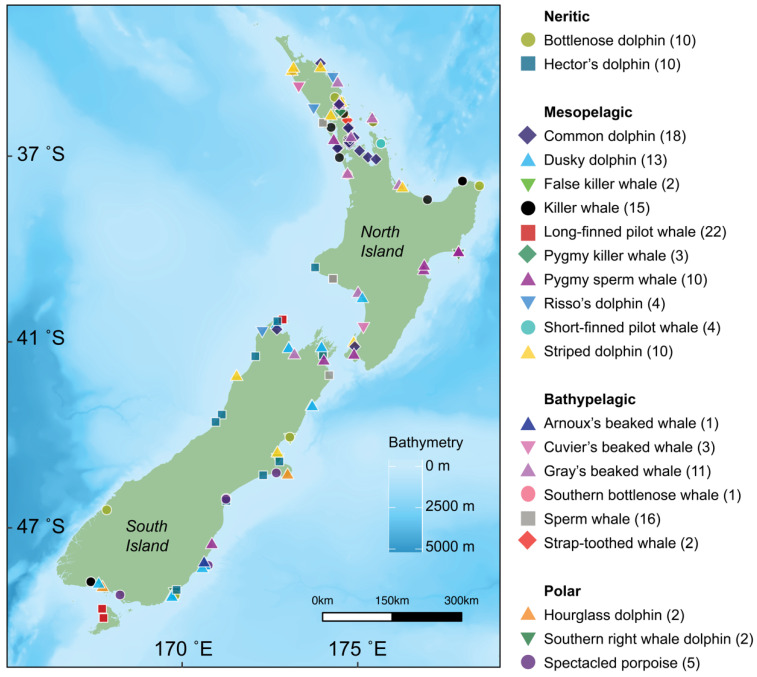
Locations for 21 species of cetaceans (denoted by shape and colour) sampled in New Zealand between 2010 and 2021. Sample size for each species is denoted in parentheses. Bathymetry is depicted with darker shades of blue representing deeper waters (data from the National Institute of Water and Atmospheric Research (NIWA) under a CC BY license, with permission from NIWA original copyright [33]).

We collected skin samples originating from live strandings or fresh to mild beach-cast events (herein collectively referred to as ‘stranded’) around the New Zealand coast (41°18′ S, 174°47′ E; Figure 1) between 2010 and 2021. Any carcass believed to be less than 24 h old, as determined by the presence of rigour mortis, the condition of the skin, and the turgor, clarity, and moisture of the eye, was defined as ‘fresh’ (codes 1 & 2 from IJsseldijk, et al. [30]). Carcasses with early stages of visible decomposition, including eyes and skin degradation, were considered ‘mild autolysis/decomposition’ (code 3) [30,31]. Animals were sexed anatomically or genetically (see Appendix A.1 for details). Sampling of both sexes occurred across independent animals only (i.e., individuals deemed to be maternally independent based on species-specific total body length as outlined by Jefferson, et al. [32]). As ontogenetic diet shifts affect isotopic values, we conservatively excluded any animals whose total body length did not fall clearly within the expected range of an independent animal from this study. We systematically sampled skin tissue comprising the complete epidermal layer from stranded carcasses and stored them in 95% ethanol or frozen at −20 °C upon collection [31].

We analysed samples from a total of 21 species, representing a wide diversity of habitats [19], represented by five families (Ziphiidae, Delphinidae, Physeteridae, Kogiidae, and Phocoenidae, Table 1).

### 2.2. Stable Isotope Analysis

We used δ^13^C and δ^15^N data as proxies for habitat and trophic position, respectively. We analysed skin samples from 164 individuals across 21 odontocete species (Table 1, Figure 1). Skin samples stored in ethanol were placed under a fume hood or a stream of nitrogen gas until the ethanol had fully evaporated. Frozen samples were slowly defrosted at room temperature. Using a stainless-steel scalpel, we cut each sample into about 0.2 mm fine slices, equivalent to approximately 10 mg of skin, and then oven dried them for at least 48 h. 

We sealed 0.5–1.0 mg of dried homogenised sample into tin capsules, which were subsequently analysed using a DELTA V Plus continuous flow isotope ratio mass spectrometer linked to a Flash 2000 elemental analyser with a MAS 200 R autosampler (Thermo Fisher Scientific, Bremen, Germany), as detailed in Peters, et al. [55]. Repeat analysis of international NIST standards produced data accurate to within or better than 0.15‰ for both δ^13^C and δ^15^N, and a precision of better than 0.24‰ for δ^13^C and 0.22‰ for δ^15^N.

Isotopic ratios were calculated as:

(1) $$\delta Χ=\left[\left(R_{\mathrm{sample}}/R_{\mathrm{standard}}\right)-1\right]\times 1000$$

where Χ is ^13^C or ^15^N, and R_sample_ and R_standard_ are the ^13^C/^12^C and ^15^N/^14^N ratios in the sample and standard, respectively. See Peters, et al. [55] for more details on the analytical protocol.

### 2.3. Lipid Extraction

Cetacean skin is known to have a high lipid content [16,56,57], which can lead to decreased δ^13^C values due to the ^12^C enrichment in the lipids [58]. Several of our bulk isotope sample analyses had a mass ratio of carbon and nitrogen (C:N) > 3.5, indicating lipid content of the tissue and thus lipid “contamination” of the carbon isotope value [59,60,61]. To account for the effect of lipid content on δ^13^C values, tissue samples need to either be lipid-extracted chemically *a priori*, or results need to be mathematically corrected *a posteriori* (normalisation). A combination of these two methods can also be applied by chemically analysing a subset of the samples to then develop a species-specific mathematical correction formula, which has cost-benefits compared to chemical lipid extraction of all samples.

To develop a mathematical normalisation formula specific to our study species, we lipid-extracted a subset of 74 samples across species, selected to cover the range of measured C:N mass ratio values (from 3.0 to 8.6). Freeze-dried material was sub-sampled and wrapped in GF/C filters prior to lipid extraction on a DIONEX 200 accelerated solvent extraction system (ASE). Samples were transferred to 22 mL s/s ASE cells and extracted three times with dichloromethane at 70 °C and 1500 psi for a static hold time of five minutes. All samples were heated to 40 °C in an oven overnight following extraction, to evaporate off any traces of solvent prior to isotope analysis.

We calculated a δ^13^C lipid-correction factor using averaged bootstrapped linear regression analysis of the δ^13^C values of the original bulk (non-lipid-extracted) and lipid-extracted samples (see Appendix A.2 for details and correction formulae). Using this factor, we mathematically corrected non-lipid extracted δ^13^C values for samples with C:N mass ratios > 3.5. For samples with C:N mass ratios < 3.5, we used the original bulk non-lipid corrected δ^13^C values. As lipid extraction can affect δ^15^N values [62,63,64], we used the non-lipid extracted δ^15^N values for all samples.

Since the samples were collected over a span of twelve years (2010–2021), we applied a correction of –0.022‰ year^−1^ [65] referenced to the year 2021 to carbon isotope values of all samples to account for the “Suess effect” (changes in the δ^13^C values of atmospheric carbon dioxide (CO^2^) due to the burning of fossil fuels) [65,66].

### 2.4. Analysis

**Figure 2:**
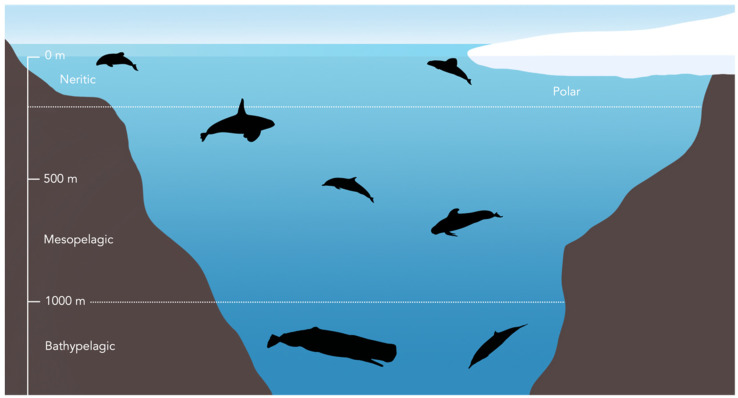
Habitat zones in which exemplar odontocete species are grouped (see Table 1). Note that not all species included in the study are shown here.

We assigned all species to one of four groups based on their most common known distribution and habitat in New Zealand waters see Table 1 in [19]: neritic habitat (bottlenose dolphin *Tursiops truncatus*, Hector’s dolphin *Cephalorhyncus hectori hectori*); mesopelagic habitat (common dolphin *Delphinus delphis*, dusky dolphin *Lagenorhynchus obscurus*, false killer whale *Pseudorca crassidens*, killer whale *Orcinus orca*, long-finned pilot whale *Globicephala melas edwardii*, pygmy killer whale *Feresa attenuata*, pygmy sperm whale *Kogia breviceps*, Risso’s dolphin *Grampus griseus*, short-finned pilot whale *G. macrorhynchus*, striped dolphin *Stenella coeruleoalba*); bathypelagic habitat (Arnoux’s beaked whale *Berardius arnuxii*, Cuvier’s beaked whale *Ziphius cavirostris*, Gray’s beaked whale *Mesoplodon grayi*, southern bottlenose whale *Hyperoodon planifrons*, sperm whale *Physeter macrocephalus*, strap-toothed whale *Mesoplodon layardii*); and polar habitat (hourglass dolphin *L. cruciger*, southern right whale dolphin *Lissodelphis peronii*, spectacled porpoise *Phocoena dioptrica*) (Table 1, and see Figure 2 for illustration of habitat zones). We calculated the mean and standard deviation of δ^15^N and δ^13^C values for all species except for Arnoux’s beaked and southern bottlenose whales, for which only one specimen per species was available.

Ten species had *n* ≥ 10 (Table 1), allowing for statistical analyses. We compared mean δ^13^C and δ^15^N values between these species using a two-sample randomisation test with 10,000 permutations at a 0.05 level. This test compares the difference of the mean isotopic values between two species with the difference obtained by randomly allocating the values among the two species [67]. Randomisation tests do not assume normal distribution or homogeneity of variances and hence are an excellent test to use for small sample sizes and biological data [67]. 

While the remaining 11 species had sample sizes <10, we have included their data in a descriptive sense as, due to the rarity of these samples for most of these cetaceans, their results certainly hold value. For these species, our data were often biased towards males. For most species with *n* ≥ 10, data were reasonably balanced between sexes, except for sperm whales, which were all male. However, overall sample sizes were too low to investigate sex-specific differences within species.

We used six different Layman metrics (δ^13^C range, δ^15^N range, total area (TA), mean distance to centroid (CD), mean nearest neighbour distance (MNND), and standard deviation of nearest neighbour distance (SDNND) to compare isotopic niches between species (see Appendix A.3 for metric definitions). All Layman metrics were bootstrapped with replacement (*n* = 10,000, indicated with a subscript ‘_boot_’) based on the smallest sample size in the data set (neritic: *n* = 10; pelagic: *n* = 10; bathypelagic: *n* = 11) to enable statistical comparison between species [67,68]. To further assess niche widths and isotopic niche overlap for each species, we followed a Bayesian approach using multivariate ellipse-based metrics [69]. This method is particularly useful when comparing groups with small sample sizes, as it corrects for the influence of outliers. We calculated standard ellipse areas (SEA), which are the bivariate equivalent to standard deviation in univariate analyses, and further calculated SEA corrected (SEA_C_) to minimise bias introduced by small sample sizes. In addition, we calculated the Bayesian SEA (SEA_B_) using 1000 posterior draws to statistically compare niche width between species. The SEA_B_ was used to calculate the niche overlap between species of the same habitat group, which was calculated as the proportion of the total SEA_B_ for each species, respectively. All statistical analyses were done using R version 4.1.0 [70]. We calculated Layman metrics and SEAs with the R package SIBER (Stable Isotope Bayesian Ellipses in R [69]).

## 3. Results

**Figure 3:**
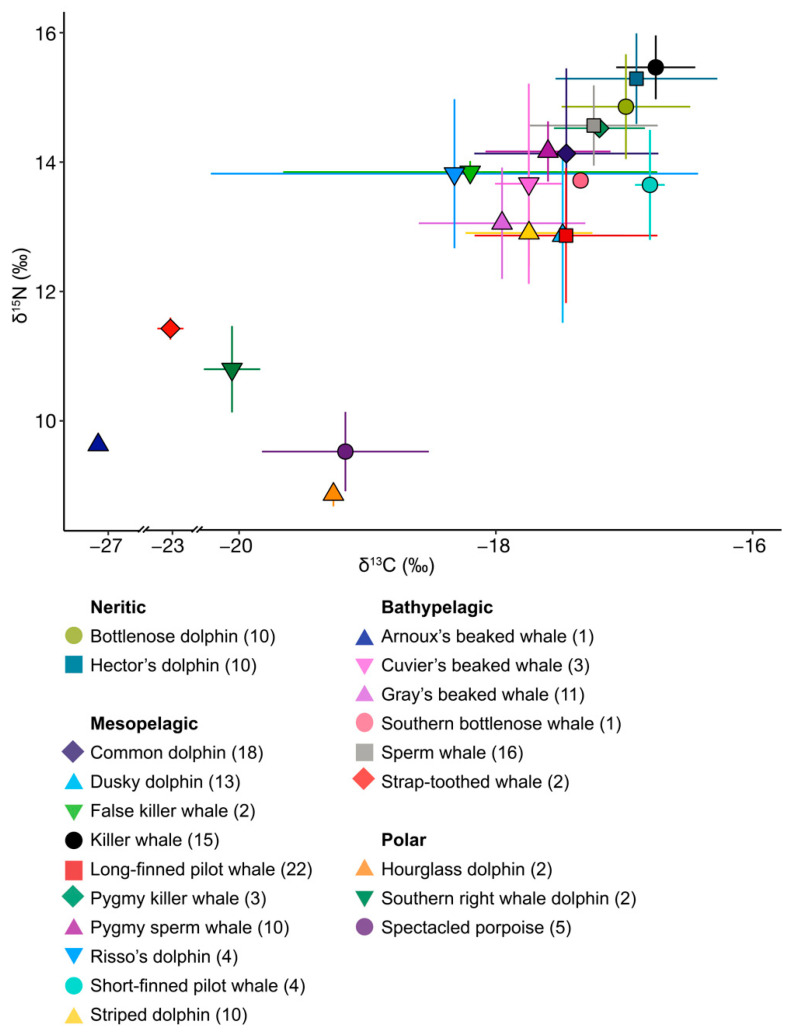
Mean ± 1 standard deviation (SD) of carbon and nitrogen isotope values for odontocete species (denoted by shape and colour) stranded in New Zealand between 2010 and 2021 (for details, see Table 1, for individual values, see Figure A1). Note that the *x*-axis is split for display purposes.

Mean isotopic values for both δ^13^C and δ^15^N differed between the 10 species with *n* ≥ 10 (randomisation test, Table 2 and Table A1, Figure 3), with all species showing differences in either δ^13^C or δ^15^N values or both compared to at least seven of the remaining nine species. Of all the pairings, 52.5% (21/40) differed in both δ^13^C and δ^15^N values, 7.5% of pairings (3/40) differed only in δ^13^C values, and 25.0% (10/40) differed only in δ^15^N values.

### 3.1. Neritic Group

**Figure 4:**
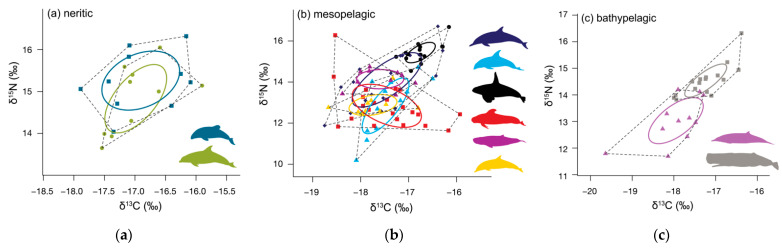
Standard ellipse area corrected for small sample size (SEA_c_, solid lines) and convex hull area (TA, dotted line) for adults. (**a**) neritic odontocetes: bottlenose dolphins (*Tursiops truncatus*, olive green), Hector’s dolphins (*Lagenorhynchus obscurus*, teal), (**b**) mesopelagic odontocetes: common dolphins (*Delphinus delphis*, dark blue), dusky dolphins (*Lagenorhynchus obscurus*, light blue), killer whales (*Orcinus orca*, black), pygmy sperm whales (*Kogia breviceps*, magenta), long-finned pilot whales (*Globicephala melas*, red), and striped dolphins (*Stenella coeruleoalba*, yellow), and (**c**) bathypelagic odontocetes: Gray’s beaked whales (*Mesoplodon grayi*, purple) and sperm whales (*Physeter macrocephalus*, grey). Ellipse areas hold 40% of the data.

**Figure 5:**
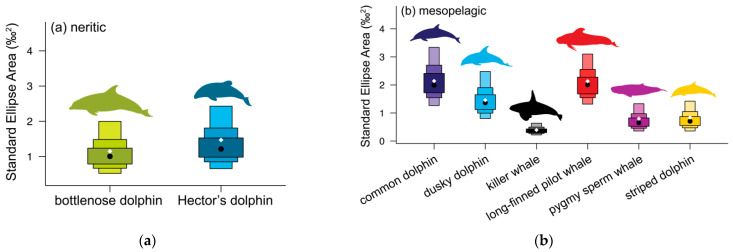

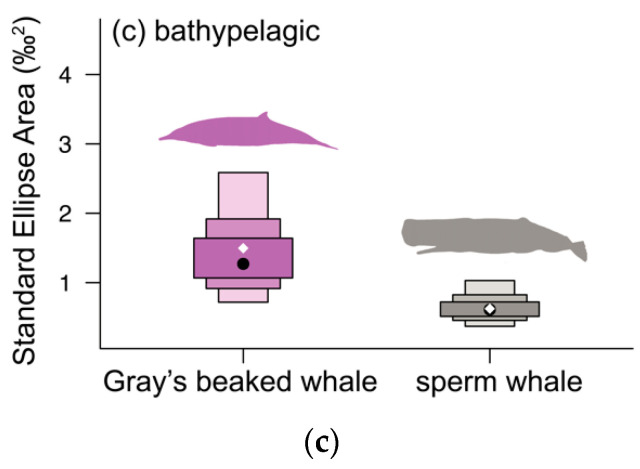
Density plot showing the 95, 75, and 50% credible intervals of standard ellipses area using Bayesian techniques for (**a**) neritic odontocetes: bottlenose dolphins (*Tursiops truncatus*), Hector’s dolphins (*Lagenorhynchus obscurus*), (**b**) mesopelagic odontocetes: common dolphins (*Delphinus delphis*), dusky dolphins (*Lagenorhynchus obscurus*), killer whales (*Orcinus orca*), pygmy sperm whales (*Kogia breviceps*), long-finned pilot whales (*Globicephala melas edwardii*), and striped dolphins (*Stenella coeruleoalba*), and (**c**) bathypelagic odontocetes: Gray’s beaked whales (*Mesoplodon grayi*) and sperm whales (*Physeter macrocephalus*). Black dots represent the mean standard ellipses area (SEA) for each species; white diamonds indicate the corrected standard ellipses area (SEA_C_).

Bottlenose and Hector’s dolphins did not differ in their mean isotopic values for either δ^13^C or δ^15^N (randomisation test: δ^13^C *p* = 0.373, δ^15^N *p* = 0.106, Table 2 and Table A1, Figure 3 and Figure A2a). Hector’s dolphins exhibited a larger SEA_B_ compared to bottlenose dolphins. However, the probability for Hector’s dolphins to have higher values for the isotopic metrics considered was low (35.3–68.0%) except for the δ^13^C range (76.1%, Table 3a). Their SEA_B_s overlapped substantially (bottlenose dolphin = 41.0%, Hector’s dolphin = 34.0%, Table 4, Figure 4a and Figure 5a).

### 3.2. Mesopelagic Group

Common dolphins had lower δ^13^C and δ^15^N mean isotopic values than killer whales, and higher δ^15^N values than long-finned pilot whales and striped dolphins (randomisation test, *p* = ≤ 0.007, Table 2 and Table A1). Of the six mesopelagic species analysed, common dolphins had the largest SEA_C_ and SEA_B_ (Table 3b), and they were more likely to have higher bootstrapped values in most Layman metrics than killer whales and striped dolphins, and in all Layman metrics compared to pygmy sperm whales (Table 5). SEA_B_ overlap for common dolphins was highest with killer whales (50.0%), smallest with striped dolphins (11.0%) and intermediate with the remaining pelagic species (19.0–27.0%) (Table 4). 

Dusky dolphins, together with long-finned pilot whales, had the lowest mean δ^15^N values of all mesopelagic species (12.84 ± 1.34‰, Table 1, Figure 3 and Figure A2c). They differed from common dolphins in both δ^13^C and δ^15^N mean isotopic values (randomisation test, *p* = 0.001, Table 2 and Table A1), but not compared to long-finned pilot whales and striped dolphins. For all Layman metrics except SDNND, dusky dolphins were more likely to have higher bootstrapped values compared to killer whales, pygmy sperm whales, and striped dolphins (Table 5). They showed high SEA_B_ overlap with long-finned pilot whales (54%) and moderate overlap with common dolphins, striped dolphins, and pygmy sperm whales (22.0–26.0%) (Table 4).

Killer whales had lower mean δ^13^C values (−16.75 ± 0.31‰) and higher mean δ^15^N values (15.43 ± 0.49‰, Table 1, Figure 3 and Figure A2c) than the other six mesopelagic species analysed (randomisation test, all *p* ≤ 0.001 Table 2 and Table A1). Killer whales were also likely to have smaller values for almost all Layman metrics compared to the other mesopelagic species (Table 5). Their SEA_C_ was the smallest compared to the other mesopelagic species, but this was not reflected in their SEA_B_. Killer whales overlapped in SEA_B_ only with common dolphins (30.0%, Table 4).

Long-finned pilot whales, together with dusky dolphins, had the lowest δ^15^N values of the six mesopelagic species analysed (12.84 ± 1.04‰, Table 1, Figure 3 and Figure A2c). This difference was significant when compared to killer whales (for both δ^13^C and δ^15^N values), and to common dolphins and pygmy sperm whales (for δ^15^N, randomisation test, *p* ≤ 0.001, Table 2 and Table A1). Long-finned pilot whales had the second highest SEA_C_ and were likely to have higher bootstrapped values in most Layman metrics compared to killer whales, pygmy sperm whales, and striped dolphins (Table 5). They overlapped in SEA_B_ with dusky dolphins (39.0%), common dolphins (28.0%), and striped dolphins (24.0%) (Table 4).

Pygmy sperm whales had the second lowest δ^13^C value (−17.59 ± 0.49‰) and the second highest mean δ^15^N value (14.14 ± 0.46‰) of the six mesopelagic species analysed (Table 1, Figure 3 and Figure A2c). They had lower mean isotopic values for both δ^13^C and δ^15^N compared to killer whales, but higher mean δ^15^N values than dusky dolphins, long-finned pilot whales, and striped dolphins (randomisation test, *p* ≤ 0.007, Table 2 and Table A1). They were likely to have lower values for most Layman metrics than common and dusky dolphins, as well as long-finned pilot whales (Table 5). Although pygmy sperm whales had the second smallest SEA and SEA_C_, this was not reflected in their SEA_B_ (Figure 5b). They showed high SEA_B_ overlap with common dolphins (both 64%) and moderate overlap with long-finned pilot whales (18%) and dusky dolphins (15%, Table 4).

Striped dolphins had lower mean δ^13^C and δ^15^N values compared to killer whales’ mean isotopic values (randomisation test, *p* < 0.001, Table 2 and Table A1) and lower mean δ^15^N values than common dolphins and pygmy sperm whales (randomisation test, *p* ≤ 0.003, Table 2 and Table A1). They were likely to have lower values for most Layman metrics than long-finned pilot whales, common, and dusky dolphins (Table 5). Striped dolphins had the second smallest SEA_C_ and SEA_B_ (Table 3b, Figure 5b), overlapping in SEA_B_ with long-finned pilot whales (63.0%), dusky dolphins (42.0%), and common dolphins (31.0%) (Table 4). 

The mean isotopic values of false killer whales, pygmy killer whales, Risso’s dolphins, and short-finned pilot whales fell within the δ^13^C and δ^15^N ranges of the other mesopelagic species. Notably, Risso’s dolphins (*n* = 4) had a large SD for δ^13^C (1.9‰).

### 3.3. Bathypelagic Group

Gray’s beaked whales had lower mean δ^13^C and δ^15^N values than sperm whales (Gray’s beaked whales δ^13^C −17.95 ± 0.65‰, δ^15^N 13.03 ± 0.86‰, sperm whales δ^13^C−17.24 ± 0.5‰, δ^15^N 14.54 ± 0.62‰, randomisation test: *p* ≤ 0.001, Table 1, Table 2 and Table A1, Figure 3 and Figure A2e). Gray’s beaked whales had higher values than sperm whales for all Layman metrics except bootstrapped MNND (Table 3c). The probability of Gray’s beaked whales having higher values was high for SEA_B_ (98.0%) and CD (86.6%), and low to moderate for all other values (64.1–78.8%, Table 3c, Figure 5c). Both species barely overlapped in their respective SEA_B_s (Gray’s beaked whales = 4.0%, sperm whales = 9.0%, Table 4, Figure 4c and Figure 5c).

**Figure A1:**
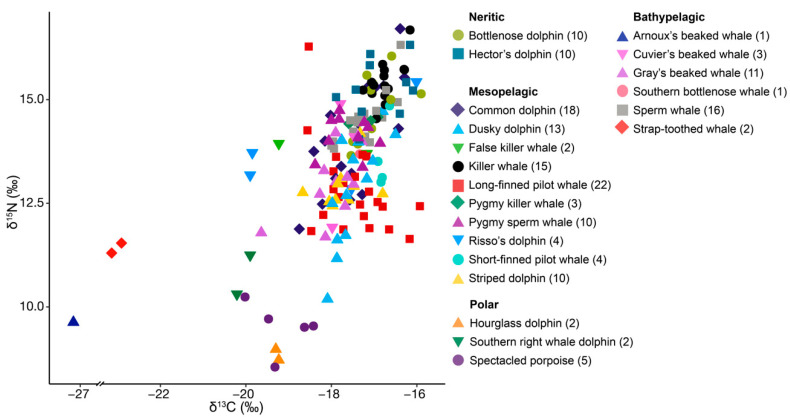
Isotopic values for individual odontocetes (species denoted by shape and colour) stranded in New Zealand between 2010 and 2021 (Table 1). Note that the *x*-axis is split for display purposes.

Mean values for Cuvier’s beaked whales and the single values for southern bottlenose whales aligned with Gray’s beaked whales. Arnoux’s beaked whale had the lowest δ^15^N value of all species in the bathypelagic group (9.6‰). Furthermore, of all the samples analysed here, Arnoux’s beaked whale had by far the lowest δ^13^C value (−27.16‰), followed by the two samples of strap-toothed whales (mean δ^13^C = −23.03 ± 0.16‰, Table 1, Figure A1).

### 3.4. Polar Group

**Figure A2:**
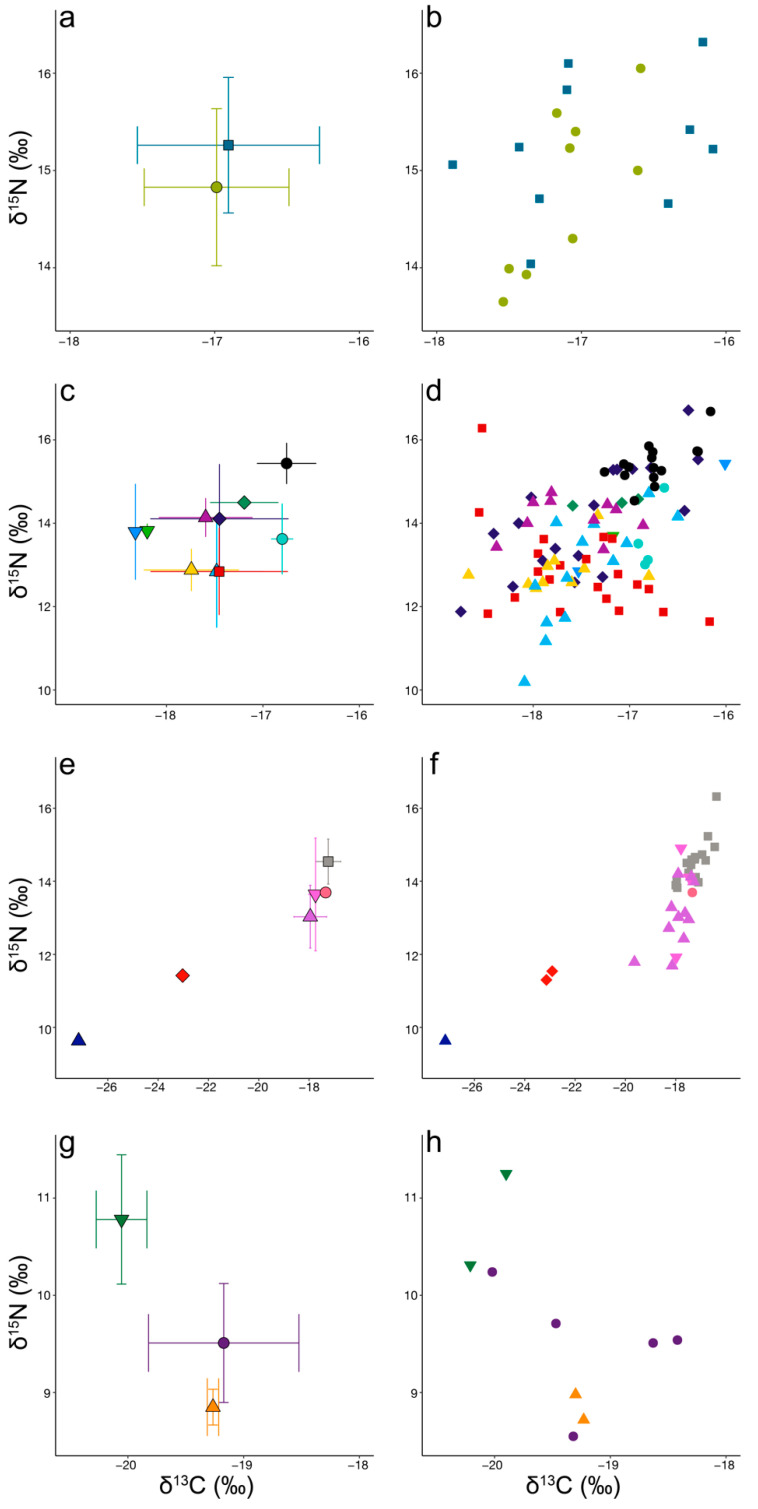
Isotopic values for individual odontocetes stranded in New Zealand between 2010 and 2021 displayed by habitat (Table 1) for (**a**,**b**) neritic odontocetes, (**c**,**d**) mesopelagic odontocetes, (**e**,**f**) bathypelagic odontocetes, and (**g**,**h**) polar odontocetes. Species denoted by shape and colour, (for legend see Figure A1).

Of all the species in this study, hourglass dolphins and spectacled porpoises had the lowest mean δ^15^N value, (8.85 ± 0.18 and 9.51 ± 0.61‰, respectively). Of the three odontocetes in the polar group, the two southern right whale dolphins had the lowest mean δ13C value (−20.06 ± 0.22‰) and the highest mean δ^15^N (10.78 ± 0.66‰, Table 1, Figure 3 and Figure A2g).

## 4. Discussion

Understanding how species navigate interspecific competition is a challenge across practically all ecological spheres. However, when dealing with some of the most poorly understood cryptic bathypelagic species, such as beaked whales, for which almost nothing is known about their ecology, this challenge is even greater. In this study, we show differing levels of isotopic niche partitioning among 21 odontocete species inhabiting three different habitats (neritic, meso-, and bathypelagic) within New Zealand waters. Our findings demonstrate the considerable overlap of stable isotopic niches among species, highlighting the need for extended assessment of foraging ecology between key overlapping species using multiple dietary markers to ascertain the full extent of overlap using complimentary methods of diet assessment. Furthermore, we use the most common feeding habitat for each species to compare species within each habitat. It is possible that a portion of feeding behaviour takes place in other habitats as well.

### 4.1. Neritic Group

Bottlenose and Hector’s dolphins, which both inhabit New Zealand’s neritic waters, demonstrated high isotopic overlap and little evidence for trophic segregation. For Hector’s dolphins, stomach content analyses indicate a diet of predominantly small and often juvenile fish and squid from throughout the water column [71]. Like many small delphinids, Hector’s dolphins appear to be relatively opportunistic feeders. Typically, their diets reflect prey species availability, which differs between regions [71], and may also be affected by opportunistic feeding, such as observed feeding behind trawlers [72].

Bottlenose dolphins (*Tursiops truncatus*) are known to occur in New Zealand as two morphologically different ecotypes, coastal and oceanic [73,74]. In this study, we only included individuals of the coastal ecotype. The diet of bottlenose dolphins globally varies between locations and populations, with a variety of fish and squid species described [34]. Isotopic values for bottlenose dolphin skin collected from free-ranging individuals in Doubtful Sound, South Island, New Zealand, yielded similar isotopic values to those presented here (δ^13^C = –16.5 ± 0.38‰, δ^15^N = 15.1 ± 0.36‰, *n* = 11, [75]). Results suggested that the local dolphin population mainly feeds on reef-associated and demersal fishes [75]. 

Given their shared use of coastal waters and similar foraging ecology as opportunistic generalists, we expected Hector’s and bottlenose dolphins to differ in their respective isotopic niches as a method of avoiding resource competition, which does not seem to be the case. As it is possible for different food resources to show similar isotopic composition [76,77], we cannot completely exclude the possibility of trophic segregation based on stable isotope data alone. Two coastal delphinid species in Queensland, Australia, the Australian humpback (*Sousa sahulensis*) and the snubfin dolphin (*Orcaella heinsohni*), also showed high isotopic niche overlap [78]. In this case, their co-existence was attributed to subtle differences in habitat use and prey selection [78]. However, those two species are similar in size and can exploit the same prey species. In the case of Hector’s and bottlenose dolphins, the smaller mouth gape makes it highly unlikely for Hector’s dolphins to be able to feed on adults of fish species such as mullet and snapper, which have been documented as prey species of bottlenose dolphins (Massey University unpublished data). However, it is possible that Hector’s dolphins feed on juveniles of the same prey species as bottlenose dolphins. Furthermore, there may be spatial or temporal segregation between the species, which reduces direct competition and thus enables their coexistence. 

### 4.2. Mesopelagic Group

Odontocetes in the mesopelagic group varied widely in niche space and Layman metrics, although we noted considerable overlap between species. For example, common dolphins exhibited the largest isotopic niche of all mesopelagic species, which reflects their generalist status, mainly feeding opportunistically on locally abundant small schooling fish or cephalopods [79,80,81]. Furthermore, rather than exclusively foraging offshore, in New Zealand the species is abundant within inshore coastal waters, such as the Hauraki Gulf [82]. Such a shallow coastal habitat likely widens the foraging niche of the population, as it includes a range of benthic species [55,79]. Indeed, common dolphins overlapped most (50%) in the Bayesian niche with killer whales, and least (11%) with striped dolphins, which is considerably smaller compared to an overlap of 45% observed with striped dolphins in the Mediterranean Sea [7].

Long-finned pilot whales demonstrated the second largest isotopic niche (Figure 4b), which overlapped with common, dusky, and striped dolphins. The large δ^13^C and δ^15^N ranges reported for this species indicate that long-finned pilot whales in New Zealand waters forage over a range of different habitats and prey of different trophic levels [83]. Based on stomach content studies, the diet of long-finned pilot whales in the Southern Ocean appears to be dominated by adult cephalopods, with local variation in the number of different species consumed [84,85,86]. Cephalopods are generally an important part of the diets of mesopelagic dolphins [87,88], which may explain the niche overlap of long-finned pilot whales with these species.

Striped dolphins demonstrated a small niche which overlapped substantially with long-finned pilot whales (63%), dusky (42%), and common dolphins (31%). Like long-finned pilot whales, striped dolphins are typically observed further offshore [19], and they likely forage in deep waters [89]. In other locations, the species is known to feed on cephalopods, fish, and secondarily on crustaceans [90,91,92]. Compared to the Mediterranean Sea, striped dolphins in New Zealand waters exhibited a larger isotopic niche [7]. While dietary similarities exist between regions, no detailed dietary information exists for New Zealand waters. A deep-water cephalopod-heavy diet would explain the high niche overlap with long-finned pilot whales.

Like common dolphins, dusky dolphins displayed a large δ^15^N range, indicating high trophic variety throughout the population, although the mean δ^15^N value was lower than in common dolphins. Dusky dolphins feed on small schooling fishes, targeting some of the same species as common dolphins, such as anchovy (*Engraulis australis*), garfish (*Hyporhamphus ihi*), and pilchard (*Sardinops neopilchardus*) [79,93,94]. However, although generally using the same mesopelagic habitat type throughout their range in New Zealand waters, dusky and common dolphins rarely occur in the same location [95], which may allow overlap in prey species. Niche overlap between dusky dolphins and common dolphins was not as high as between dusky dolphins and long-finned pilot whales (26% vs. 63%).

Pygmy sperm whales signalled a small niche area and δ^15^N range, and their Layman values were likely to be lower compared to the other mesopelagic species, except killer whales. Despite the samples having been collected in five different years between 2009 and 2021, pygmy sperm whales had a small isotopic niche, indicating little diversity in basal resources. Similarly, to long-finned pilot whales, the diet of pygmy sperm whales consists mainly of oceanic cephalopods caught in depths of up to 1100 m, with smaller proportions of fish and crustaceans [96,97,98,99]. However, niche overlap was three times as high with common dolphins (64%) than with long-finned pilot whales (18%), suggesting more competition with common dolphins. Given the greater potential foraging depth of pygmy sperm whales compared to common dolphins [79,100,101], it is likely that vertical niche stratification alleviates competition between these two species.

As expected, based on what is known about their foraging ecology as apex predators, [39], killer whales had the highest mean δ^15^N values of all species analysed here. Killer whales are generalist predators with a diet that includes both fish and marine mammals [39]. However, local populations can be highly specialised in foraging and hunting strategies, with some populations primarily feeding on fish [102,103,104] and others almost exclusively on marine mammals [102,105]. In New Zealand, killer whales are documented to regularly feed on stingrays [106] and sharks [107,108], with marine mammal predations also recorded [109]. In locations where prey specialisation occurs, stable isotope analysis has been able to quantify inter-individual dietary variation, showing higher δ^15^N values for individuals feeding on marine mammals compared to those feeding on fish [104,110,111,112]. Interestingly, the isotopic niche of killer whales in this study was the smallest of all mesopelagic species, suggesting little isotopic variety in their diet and limited inter-individual differences, which is further confirmed by the low Layman metrics values. Our killer whale data comprise samples from six stranding events (two mass strandings and four single strandings). It is possible that the eleven animals deriving from the two mass strandings (five and six animals, respectively) skew the data somewhat, particularly if the mass stranded animals comprised part of the same group and foraged in the same area and on similar prey prior to stranding.

Risso’s dolphins and false killer whales had the lowest mean δ^13^C values (−18.32 ± 1.90‰ and −18.20 ± 1.46‰, respectively) of the mesopelagic species, indicating that these animals likely foraged further offshore than the other species. While nothing is known about the diet of Risso’s dolphin in New Zealand, false killer whales are known to feed cooperatively with oceanic bottlenose dolphins on Kahawai (*Arrips trutta*) off North Island, New Zealand [113] and travel large distances across north-eastern New Zealand [114]. Contrastingly, mean δ^13^C values of pygmy killer whales (−17.19 ± 0.35‰) and short-finned pilot whales (−16.8 ± 0.11‰) suggested feeding in waters closer to shore, similar to killer whales. However, sample sizes for these four species were low, preventing isotopic niche analyses.

Overall, species in the mesopelagic group showed less niche differentiation than similar species conglomerates in other locations [5,115,116]. This suggests that competition between species is reduced through other mechanisms, such as fine-scale spatial or temporal segregation. Furthermore, the samples analysed here originate from various locations around New Zealand, which itself spans a large latitudinal range (S 34.5–47). It is possible that local populations of some species differ in their foraging ecology, which could change their isotopic overlap with local competitors. For most of the mesopelagic species analysed here, there is little information about either their diet or fine-scale distribution in New Zealand waters. Consequently, they may be feeding on abundant prey, allowing coexistence, or alternatively consuming different prey with similar isotopic compositions, which would remain undetected through bulk stable isotope analysis. Furthermore, vertical niche segregation via differences in foraging depths could also be at play.

### 4.3. Bathypelagic Group

Although sperm whales and Gray’s beaked whales are both species that forage in the deep sea, they have almost no overlap in their core isotopic niche. Due to their deep-water habitat and generally elusive nature, little is known about the foraging ecology and diet of beaked whales [51]. Prey items for species of the genus *Mesoplodon* are thought to include small mesopelagic squid, fish, and also crustaceans [117]. However, the stomachs of three Gray’s beaked whales from Brazil, South Africa, and the Indian Ocean did not contain any squid, indicating this species may feed primarily on fish [117]. While very little information is available on the diet of beaked whales in New Zealand waters, identifiable hard part remains from the stomachs of five Gray’s beaked whales included lanternfish (Myctophidae), as well as other mid-water fish and squid remains [118].

Although the New Zealand sperm whale diet is dominated by oceanic cephalopods, it also includes demersal fish [119]. As males tend to eat more demersal fish than females [50], this likely reflects the distributional bias towards males below 42° latitude [120], which is echoed in our data as all 16 animal samples were males. Sperm whales had higher δ^15^N values than Gray’s beaked whales, suggesting that they feed on larger prey. The considerable size difference between the two species (total body length range of individuals analysed here: sperm whales = 1105–1677 cm, Gray’s beaked whales = 305–490 cm) and the resulting difference in energy requirements is consistent with this assumption. Although sperm whales are known to be capable of hunting very large prey such as giant squid (*Architeuthis* spp.) [50], they predominantly feed on smaller squid (<1 m length) in New Zealand [119,121], which may still be larger than the prey consumed by the comparatively smaller Gray’s beaked whales.

While the Gray’s beaked whale samples analysed here were collected across five different years and at least six different stranding events, the sperm whale samples originated from three stranding events (two mass strandings and one single stranding) across two years. The two groups of three and twelve animals had likely been foraging in similar habitats, respectively. Therefore, it is not surprising that the Layman metrics values were generally higher for Gray’s beaked whales, reflecting higher inter-individual differences and larger niche width (Figure 5b). However, these differences could also reflect a genuine broader variety in prey consumed by Gray’s beaked whales compared to sperm whales.

Sperm whales in New Zealand exhibit subtle seasonal differences in their foraging patterns [122] and can also display different foraging strategies depending on their location [123]. Our data were most similar to the mean isotopic values observed in Kaikōura, New Zealand, in the winter months [122], which matches the temporal distribution (May–July) of 13 out of the 16 sperm whale samples included in this study. The remaining group of three sperm whales had lower δ^15^N and δ^13^C values, suggesting that they likely foraged elsewhere. Interestingly, their isotopic values were closer to those of the Gray’s beaked whales, suggesting that niche overlap between these two species could be higher in some locations than observed here.

Gray’s beaked whales stranded along the coast of Tierra del Fuego, Argentina, showed 15% niche overlap with Cuvier’s beaked whales [124]. The three Cuvier’s beaked whales included here had similar isotopic values to Gray’s beaked whales as well as the southern bottlenose whale. While little is known about the southern bottlenose whale diet, stomach contents of stranded individuals suggest that they mainly feed on cephalopods [125], similar to northern bottlenose whales (*H. ampullatus*) [126,127,128]. While we did not have a sufficient sample size to allow for isotopic niche comparison for all beaked whale species presented here, mean isotopic values of δ^15^N and δ^13^C did indicate some degree of niche overlap between Gray’s and Cuvier’s beaked whales, as well as southern bottlenose whales too.

Conversely, the two strap-toothed beaked whales demonstrated lower mean δ^15^N values compared to the other beaked whales, except for the Arnoux’s beaked whale, indicating they likely feed on small prey. Most cephalopods found in the stomachs of strap-toothed beaked whales in New Zealand ranged between 20–100 g and were approximately 15 cm in length, matching the species’ small gape caused by fully erupted teeth in adults, and thus their adaptation to relatively small prey [129]. Prey items to date identified include bathypelagic squid including Chiroteuthidae, Cranchiidae, and Histioteuthidae [118]. The low δ^15^N values observed for the Arnoux’s beaked whale and the two strap-toothed beaked whales included here indicate some level of resource partitioning among species, which could alleviate interspecific competition. However, the low δ^15^N values also suggest that these animals have been feeding in Antarctic or subantarctic waters, which may have exposed them to different prey than the other bathypelagic species.

### 4.4. Polar Group

Hourglass dolphins have been observed feeding in surface waters along the Antarctic convergence [52], and their diet likely includes fish and squid [130]. Similarly, southern right whale dolphins mainly occur between the subtropical and the Antarctic convergences where they feed on fish and squid [53]. Due to their remote oceanic habitat, next to nothing is known about the diet and behaviour of spectacled porpoises, although they are suspected to also forage on fish and squid in cold waters near the Antarctic convergence [54]. 

From the three species in the polar group, southern right whale dolphins had the highest δ^15^N and the lowest δ^13^C values, while spectacled porpoises and hourglass dolphins had similar isotopic values. Mean δ^13^C values for all three species ranged between −20.06 and −19.17‰, which suggests that these individuals were likely not feeding at the polar front, which would have resulted in lower δ^13^C values [131]. Based on the isotopic values of the individuals analysed here, spectacled porpoises and hourglass dolphins likely overlap in prey and foraging habitat. Stable isotopes from bone collagen collected from strandings in Tierra del Fuego, Argentina, indicated a similar pattern for the three polar species included here, with high isotopic similarity for spectacled porpoises and hourglass dolphins, and higher δ^15^N values for southern right whale dolphins [132].

To date, no consensus exists on the turnover time of cetacean skin, and estimates range from several days [133,134] to several months [135]. Given that the samples analysed here originate from animals that were stranded in New Zealand, it is possible that these individuals had already been feeding outside of their usual polar habitat for a considerable time, which means that their isotopic values may not reflect these species’ usual foraging ecology.

## 5. Conclusions

We present the first comparative isotopic niche assessment of New Zealand’s odontocetes. Aside from Gray’s beaked whales and sperm whales, which show clear niche separation, all species overlapped substantially in their isotopic niche with at least one other species in the same habitat. Our findings suggest other mechanisms must be at play to reduce interspecific competition and thus enable coexistence in New Zealand’s biodiverse waters. Many of the species included here are unlikely to be directly observed while foraging owing to their elusive nature, offshore/deep-water habitat and/or foraging behaviour. Accordingly, future studies would benefit from a combined approach that extends bulk stable isotope analyses and includes fatty-acid and compound-specific stable isotopes alongside to provide a deeper understanding of the diet of these species. Where relevant, these analyses would support stomach content studies, which could further be assisted using metabarcoding of prey. Furthermore, bulk stable isotopes of hydrogen, sulphur, and oxygen may provide increased resolution on habitat use and resource pathways [136,137,138]. Since interspecific competition only occurs when the resource that the species compete for is limited [23,24], studies are required to examine the extent of species competition by assessing prey abundance, fine-scale habitat use, foraging depths, and differences between local populations for odontocete species in New Zealand. While this study clearly highlights the many remaining unknowns in foraging ecology and potential interspecific competition of New Zealand’s odontocete community, it presents an important foundational step, particularly considering current and future ecosystem changes such as ocean warming and fishery pressures causing alterations to prey abundance and distributions.

## Figures and Tables

**Table 1:** Suess-corrected carbon (δ^13^C) and nitrogen (δ^15^N) isotope values and sample sizes (*n*) for independent odontocete species sampled in Aotearoa, New Zealand, 2010–2021. Values are given as mean ± 1 SD. See Figure 2 for an illustration of habitat zones.

Group	Species	Latin Name	Preferred Habitat	*n*	δ^13^C	δ^15^N
**Neritic**	Bottlenose dolphin	*Tursiops* *truncatus*	Temperate coastal to pelagic waters [34]	10	−16.99 ± 0.50	14.83 ± 0.81
	Hector’s dolphin	*Cephalorhyncus hectori hectori*	Coastal waters [35]	10	−16.91 ± 0.63	15.26 ± 0.70
**Meso-** **pelagic**	Common dolphin	*Delphinus* *delphis*	Coastal waters [36]	18	−17.45 ± 0.72	14.11 ± 1.31
	Dusky dolphin	*Lagenorhynchus* *obscurus*	Neritic waters above continental shelves [37]	13	−17.48 ± 0.48	12.84 ± 1.34
	False killer whale	*Pseudorca* *crassidens*	Pelagic waters [38]	2	−18.20 ± 1.46	13.82 ± 0.17
	Killer whale	*Orcinus orca*	Coastal to offshore, tropical to polar waters [39]	15	−16.75 ± 0.31	15.43 ± 0.49
	Long-finned pilot whale	*Globicephala* *melas edwardii*	Cold temperate oceanic to shelf waters [40]	22	−17.45 ± 0.71	12.84 ± 1.04
	Pygmy killer whale	*Feresa attenuata*	Tropical oceanic waters [41]	3	−17.19 ± 0.35	14.50 ± 0.08
	Pygmy sperm whale	*Kogia breviceps*	Pelagic waters [42]	10	−17.59 ± 0.49	14.14 ± 0.46
	Risso’s dolphin	*Grampus griseus*	Offshore waters [43]	4	−18.32 ± 1.90	13.80 ± 1.15
	Short-finned pilot whale	*G. macrorhynchus*	Tropical to warm temperate, oceanic to shelf waters [40]	4	−16.8 ± 0.11	13.62 ± 0.85
	Striped dolphin	*Stenella* *coeruleoalba*	Waters outside the continental shelf, oceanic [44]	10	−17.74 ± 0.49	12.88 ± 0.51
**Bathy-** **pelagic**	Arnoux’s beaked whale	*Berardius arnuxii*	Southern hemisphere waters, between 24°S and Antarctica [45]	1	−27.16	9.63
	Cuvier’s beaked whale	*Ziphius* *cavirostris*	Oceanic deep waters [46]	3	−17.74 ± 0.26	13.64 ± 1.54
	Gray’s beaked whale	*Mesoplodon grayi*	Temperate deep waters [47,48]	11	−17.95 ± 0.65	13.03 ± 0.86
	Southern bottlenose whale	*Hyperoodon* *planifrons*	Deep waters south of 30°S [49]	1	−17.34	13.69
	Sperm whale	*Physeter* *macrocephalus*	Deep waters, tropical to sub-polar [50]	16	−17.24 ± 0.50	14.54 ± 0.62
	Strap-toothed whale	*Mesoplodon* *layardii*	Temperate and subantarctic waters in the Southern Hemisphere [51]	2	−23.03 ± 0.16	11.42 ± 0.17
**Polar**	Hourglass dolphin	*L. cruciger*	Pelagic-oceanic, polar waters [52]	2	−19.27 ± 0.05	8.85 ± 0.18
	Southern right whale dolphin	*Lissodelphis* *peronii*	Circumpolar subantarctic and cool-temperate Southern Ocean waters [53]	2	−20.06 ± 0.22	10.78 ± 0.66
	Spectacled porpoise	*Phocoena* *dioptrica*	Cool temperate, sub-Antarctic, and Antarctic oceanic waters [54]	5	−19.17 ± 0.65	9.51 ± 0.61

**Table 2:** Pairwise comparisons (randomisation test) of δ^13^C and δ^15^N isotopic values between odontocete species with *n* ≥ 10 within each habitat group. Colour denotes the level of difference (at 0.05 significance level): dark blue = difference in both δ^13^C and δ^15^N isotopic values, light blue: difference only in δ^15^N isotopic values, white = no difference. Neritic group (yellow): BD = bottlenose dolphin, HD = Hector’s dolphin, mesopelagic group (orange): CD = common dolphin, DD = dusky dolphin, KW = killer whale, LPW = long-finned pilot whale, PSW = pygmy sperm whale, SD = striped dolphin, bathypelagic group (green): GW = Gray’s beaked whale, SBW = southern bottlenose whale, SW = sperm whale. Dark grey indicates matrix diagonal, light grey fields refer to species not in the same habitat. See Table A1 for *p*-values.

	Neritic		Mesopelagic						Bathypelagic	
Species	BD	HD	CD	DD	KW	LPW	PSW	SD	GW	SW
**BD**										
**HD**										
**CD**										
**DD**										
**KW**										
**LPW**										
**PSW**										
**SD**										
**GW**										
**SW**										

**Table 3:** Isotopic niche metrics (including the six Layman metrics) for adult odontocetes with *n* ≥ 10. SEA = standard ellipse area, SEA_C_ = standard ellipse area corrected for small sample size, SEA_B_ = Bayesian SEA, TA = total area, CD = mean distance to centroid, MNND = mean nearest neighbour distance, SDNND = standard deviation of nearest neighbour distance, see supplementary material for metric definitions. The subscript ‘boot’ indicates that the value (mean) has been generated via bootstrapping. Probability refers to the probability of the respective bootstrapped metric being larger in one species over another across all draws.

(**a**) neritic odontocetes						
**Metrics**		**Bottlenose dolphin**		**Hector’s dolphin**	**Probability (%)**	
***N***		10		10		
**SEA**		1.02		1.31		
**SEA_C_**		1.15		1.47		
**SEA_B_**		0.78		0.99		
**δ^13^C range**		1.64		1.80		
**δ^13^C range_boot_**		1.35		1.59	76.1% HD > BD	
**δ^15^N range**		2.40		2.28		
**δ^15^N range_boot_**		2.10		1.93	64.7% BD > HD	
**TA**		1.76		2.52		
**TA_boot_**		1.82		2.01	59.1% HD > BD	
**CD**		0.84		0.85		
**CD_boot_**		0.78		0.78	51.1% BD > HD	
**MNND**		0.36		0.48		
**MNND_boot_**		0.33		0.35	56.6% HD > BD	
**SDNND**		0.24		0.22		
**SDNND_boot_**		0.26		0.28	56.1% HD > BD	
(**b**) mesopelagic odontocetes (for probability %, see Table 4)						
**Metrics**	**Common** **dolphin**	**Dusky** **dolphin**	**Killer whale**	**Long-finned** **pilot whale**	**Pygmy** **sperm whale**	**Striped** **dolphin**
***N***	18	13	15	22	10	10
**SEA**	2.01	1.33	0.37	2.03	0.71	0.74
**SEA_C_**	2.14	1.46	0.39	2.13	0.80	0.84
**SEA_B_**	2.98	1.14	2.00	2.48	2.67	1.58
**δ^13^C range**	2.46	1.59	1.10	2.63	1.52	1.87
**δ^13^C range_boot_**	2.03	1.32	0.88	2.08	1.29	1.44
**δ^15^N range**	4.83	4.53	2.14	4.64	1.37	1.75
**δ^15^N range_boot_**	3.63	3.68	1.46	2.93	1.21	1.33
**TA**	5.39	2.93	0.86	6.73	1.33	1.65
**TA_boot_**	4.66	3.06	0.74	3.54	1.03	1.10
**CD**	1.32	1.20	0.46	1.00	0.60	0.55
**CD_boot_**	1.24	1.14	0.45	0.98	0.56	0.53
**MNND**	0.45	0.47	0.20	0.45	0.36	0.39
**MNND_boot_**	0.60	0.48	0.23	0.53	0.25	0.26
**SDNND**	0.31	0.20	0.23	0.41	0.21	0.35
**SDNND_boot_**	0.38	0.35	0.21	0.43	0.20	0.27
(**c**) bathypelagic odontocetes						
**Metrics**		**Gray’s beaked whale**		**Sperm whale**	**Probability (%)**	
***N***		11		16		
**SEA**		1.35		0.58		
**SEA_C_**		1.50		0.62		
**SEA_B_**		1.46		1.45	98.0% GW > SW	
**SEA_B_ overlap**		0.03		0.09		
**δ^13^C range**		2.35		1.62		
**δ^13^C range_boot_**		1.81		1.43	65.7% GW > SW	
**δ^15^N range**		2.51		2.50		
**δ^15^N range_boot_**		2.33		1.81	73.7% GW > SW	
**TA**		2.98		1.51		
**TA_boot_**		2.66		1.57	78.7% GW > SW	
**CD**		0.85		0.60		
**CD_boot_**		0.85		0.62	86.6% GW > SW	
**MNND**		0.49		0.24		
**MNND_boot_**		0.36		0.31	64.1% GW > SW	
**SDNND**		0.40		0.26		
**SDNND_boot_**		0.34		0.27	65.4% GW > SW	

**Table 4:** Bayesian niche overlap (40%) for each species (left column) with each other species (top row) in their habitat group for species with *n* ≥ 10. Species abbreviations: Neritic group (yellow): BD = bottlenose dolphin, HD = Hector’s dolphin, mesopelagic group (orange): CD = common dolphin, DD = dusky dolphin, KW = killer whale, LPW = long-finned pilot whale, PSW = pygmy sperm whale, SD = striped dolphin, bathypelagic group (green): GW = Gray’s beaked whale, SW = sperm whale. Dark grey indicates matrix diagonal, light grey fields refer to species not in the same habitat.

Species	BD	HD	CD	DD	KW	LPW	PSW	SD	GW	SW
**BD**		0.41								
**HD**	0.34									
**CD**				0.19	0.50	0.27	0.22	0.11		
**DD**			0.26		>0.01	0.54	0.22	0.23		
**KW**			0.30	>0.01		>0.01	0.02	>0.01		
**LPW**			0.28	0.39	>0.01		0.06	0.24		
**PSW**			0.64	0.15	>0.01	0.18		0.05		
**SD**			0.31	0.42	>0.01	0.63	0.04			
**GW**										0.03
**SW**									0.09	

**Table 5:** The probability of each respective bootstrapped metric being larger in one species over another across all draws for mesopelagic species with *n* ≥ 10. Species abbreviations: CD = common dolphin, DD = dusky dolphin, KW = killer whale, LPW = long-finned pilot whale, PSW = pygmy sperm whale, SD = striped dolphin. Probabilities ≥ 90% are highlighted in bold.

Species	Metric	CD	DD	KW	LPW	PSW	SD
*Common dolphin (CD)*	SEA_B_		84.2	**100.0**	54.1	**99.4**	**99.1**
	δ^13^C range		**94.7**	**99.5**	44.8	**95.5**	85.6
	δ^15^N range		47.5	**99.5**	68.4	**100.0**	**99.9**
	TA		83.0	**100.0**	72.1	**100.0**	**99.8**
	CD		63.9	**100.0**	79.9	**1000**	**99.9**
	MNND		74.1	**99.3**	62.5	**99.2**	**98.2**
	SDNND		60.1	89.9	50.4	**92.8**	75.2
*Dusky dolphin (DD)*	SEA_B_	15.8		**100.0**	17.2	**95.0**	**93.1**
	δ^13^C range	5.1		89.4	8.3	56.7	41.8
	δ^15^N range	52.3		**99.4**	67.5	**100.0**	**99.5**
	TA	17.0		**99.6**	44.7	**98.8**	**96.3**
	CD	36.1		**99.9**	70.2	**99.6**	**99.2**
	MNND	25.9		**96.8**	40.9	**95.1**	**93.0**
	SDNND	39.9		83.2	43.1	88.0	66.5
*Killer whale (KW)*	SEA_B_	0.0	0.0		0.0	5.9	5.0
	δ^13^C range	0.5	10.5		0.9	7.0	10.5
	δ^15^N range	0.5	0.6		13.1	62.3	57.2
	TA	0.0	0.4		0.6	24.6	32.0
	CD	0.0	0.1		0.7	18.6	31.8
	MNND	0.7	3.2		2.7	40.6	38.1
	SDNND	10.1	16.8		16.7	53.0	33.5
*Long-finned pilot whale (LPW)*	SEA_B_	45.9	82.8	100.0		**99.2**	**99.2**
	δ^13^C range	53.9	91.6	**99.1**		**93.4**	84.2
	δ^15^N range	31.4	32.4	86.8		**97.3**	**93.1**
	TA	27.9	55.3	**99.4**		**98.4**	**95.7**
	CD	20.1	29.8	**99.3**		**98.1**	**96.7**
	MNND	37.5	59.1	**97.3**		**96.7**	**94.6**
	SDNND	49.6	56.9	83.3		86.5	69.4
*Pygmy sperm whale (PSW)*	SEA_B_	0.6	5.0	**94.0**	0.8		44.4
	δ^13^C range	4.4	43.0	**92.9**	6.5		34.8
	δ^15^N range	0.0	0.0	35.9	2.6		33.1
	TA	0.0	1.2	75.4	1.6		48.5
	CD	0.0	0.4	81.4	1.9		57.8
	MNND	0.8	4.9	59.4	3.3		46.8
	SDNND	7.2	12.0	47.0	13.5		31.2
*Striped dolphin (SD)*	SEA_B_	0.9	6.9	**95.0**	0.9	55.6	
	δ^13^C range	14.4	58.1	89.3	15.8	65.2	
	δ^15^N range	0.1	0.5	42.8	6.3	66.9	
	TA	0.2	3.7	68.0	4.3	51.5	
	CD	0.1	0.8	68.2	3.3	42.2	
	MNND	1.8	7.0	61.9	5.4	53.2	
	SDNND	24.8	33.5	66.5	30.6	68.8	

## Data Availability

Data are available at https://github.com/kjopeters/Peters-et-al-2022-Biology-Stable-Isotopes.

## Appendix A. Extended Methods

### Appendix A.1. Molecular Sexing

We extracted DNA from the skin using the Qiagen DNeasy Blood and Tissue Kits according to standard protocol. Molecular sexing was undertaken by targeting the SRY and ZFX gene loci [139]. We carried out PCR in 25 μL volumes using the MyTaq™ DNA polymerase kit (Bioline, Eveleigh, Australia), including: 0.3 μM of the primers PMSRYF [140], ZFX0582F, and ZFX0923R [141]; 0.6 μM of the primer TtSRYR [139]; and 50–75 ng of template DNA. Thermocycling entailed an initial denaturation at 92 °C for 30 s followed by 35 cycles of 94 °C for 30 seconds, 41 °C for 45 s, and 72 °C for 45 s, following the protocol as described in Rosel [139]. We ran the PCR products plus a negative control on a 2.8% agarose gel at 80 V for 1 h, to observe the separation of amplicons indicating male (two bands) or female (one band).

### Appendix A.2. Mathematical Lipid Correction

For the remaining species, we used a bootstrapping approach to derive a sample correction formula that is robust against outliers. We randomly selected a subset of 80% of all lipid-corrected samples (*n* = 74). We then used a linear regression analysis of the δ^13^C values of the original whole (non-lipid-extracted) and lipid-extracted samples in this subset. We repeated this process 1000 times and used the average coefficients from these 1000 replicates to derive our final correction formula:

(A1) $$\delta^{13}C_{\mathrm{LEC}}=0.5301486\times \delta^{13}C-7.322335$$

where δ^13^C is the non-lipid extracted value and δ^13^C_LEC_ is the mathematically corrected value. For common dolphins, we used a previously published species-specific correction formula [55] as this one performed slightly better for this species.

(A2) $$\delta^{13}C_{\mathrm{LEC}}=0.5409\times \delta^{13}C-7.4674$$

### Appendix A.3. Details on Layman Metrics

We used six different Layman metrics [69,142] to measure niche variation between different odontocete species:

(1) **δ^15^N range:** distance between the highest and lowest δ^15^N values (i.e., max δ^15^N—min δ^15^N). Measure of trophic length of the community.
(2) **δ^13^C range:** distance between the highest and lowest δ^13^C values (i.e., max δ^13^C—min δ^13^C). Estimates the diversity of basal resources.
(3) **Total area (TA):** total area of the convex hull comprising all data points. Measure of the total amount of niche space occupied and an indication of niche width.
(4) **Mean distance to centroid (CD):** average Euclidean distance of each sample to the centroid. Measure of niche width and sample spacing.
(5) **Mean nearest neighbour distance (MNND):** mean of the Euclidean distances to each sample’s nearest neighbour. Measure of the density and clustering of individuals.
(6) **Standard deviation of nearest neighbour distance (SDNND):** measure of the evenness of spatial density and the packing of individuals. Low SDNND values indicate a more even distribution of trophic niches.

**Table A1:** Results of pairwise comparisons (randomisation test) of δ^15^N and δ^13^C isotopic values between odontocete species with *n* ≥ 7. Significant values (at 0.05 level) denoted in bold. Neritic group (yellow): BD = bottlenose dolphin, HD = Hector’s dolphin, mesopelagic group (orange): CD = common dolphin, DD = dusky dolphin, KW = killer whale, LPW = long-finned pilot whale, PSW = pygmy sperm whale, SD = striped dolphin, bathypelagic group (green): GW = Gray’s beaked whale, SW = sperm whale. Dark grey indicates matrix diagonal, light grey fields refer to species not in the same habitat.

δ^15^N	BD	HD	CD	DD	KW	LPW	PSW	SD	GW	SW
δ^13^C										
**BD**		0.106								
**HD**	0.373									
**CD**				**0.007**	**0.001**	**0.001**	0.473	**0.003**		
**DD**			0.451		**<0.001**	0.494	**0.005**	0.463		
**KW**			**0.001**	**<0.001**		**<0.001**	**<0.001**	**<0.001**		
**LPW**			0.496	0.446	**0.001**		**0.001**	0.485		
**PSW**			0.286	0.294	**<0.001**	0.288		**<0.001**		
**SD**			0.134	0.104	**<0.001**	0.132	0.256			
**GW**										**<0.001**
**SW**									**0.001**	
